# Alteration in Auxin Homeostasis and Signaling by Overexpression Of PINOID Kinase Causes Leaf Growth Defects in *Arabidopsis thaliana*

**DOI:** 10.3389/fpls.2017.01009

**Published:** 2017-06-14

**Authors:** Kumud Saini, Marios N. Markakis, Malgorzata Zdanio, Daria M. Balcerowicz, Tom Beeckman, Lieven De Veylder, Els Prinsen, Gerrit T. S. Beemster, Kris Vissenberg

**Affiliations:** ^1^Integrated Molecular Plant Physiology Research, University of AntwerpAntwerp, Belgium; ^2^Faculty of Health and Medical SciencesCopenhagen, Denmark; ^3^Center for Plant Systems Biology, VIBGhent, Belgium; ^4^Department of Plant Biotechnology and Bioinformatics, Ghent UniversityGhent, Belgium; ^5^Plant Biochemistry and Biotechnology Lab, Department Of Agriculture, School of Agriculture, Food and Nutrition, University of Applied Sciences Crete – Technological Educational Institute (UASC-TEI)Heraklion, Greece

**Keywords:** auxin, cell division, cell expansion, kinematic analysis, leaf growth and development, *PINOID (PID)*, RNA-sequencing

## Abstract

In plants many developmental processes are regulated by auxin and its directional transport. PINOID (PID) kinase helps to regulate this transport by influencing polar recruitment of PIN efflux proteins on the cellular membranes. We investigated how altered auxin levels affect leaf growth in *Arabidopsis thaliana*. Arabidopsis mutants and transgenic plants with altered *PID* expression levels were used to study the effect on auxin distribution and leaf development. Single knockouts showed small pleiotropic growth defects. Contrastingly, several leaf phenotypes related to changes in auxin concentrations and transcriptional activity were observed in *PID* overexpression (*PID^OE^*) lines. Unlike in the knockout lines, the leaves of *PID^OE^* lines showed an elevation in total indole-3-acetic acid (IAA). Accordingly, enhanced DR5-visualized auxin responses were detected, especially along the leaf margins. Kinematic analysis revealed that ectopic expression of *PID* negatively affects cell proliferation and expansion rates, yielding reduced cell numbers and small-sized cells in the *PID^OE^* leaves. We used *PID^OE^* lines as a tool to study auxin dose effects on leaf development and demonstrate that auxin, above a certain threshold, has a negative affect on leaf growth. RNA sequencing further showed how subtle *PID^OE^*-related changes in auxin levels lead to transcriptional reprogramming of cellular processes.

## Introduction

For agronomical reasons and to improve our knowledge of organ size control in multicellular organisms it is important to understand the genetic basis and the encoded molecular circuitry regulating the growth of organs such as leaves (Powell and Lenhard, 2012; Nelissen et al., 2014; Czesnick and Lenhard, 2015; Vanhaeren et al., 2015). Leaf initiation occurs at the periphery of the shoot apical meristem (SAM) where a few stem cells start to proliferate and develop into a primordium, which subsequently grows out to form a leaf. These latter stages of growth are solely defined by two distinct but overlapping processes: cell proliferation that results from cell division (cell cycle activity), and subsequent cell expansion, driven by vacuolar expansion and cell wall extension through the activity of numerous cell wall modifying proteins (Green, 1976; Green and Bauer, 1977; Beemster and Baskin, 1998; Donnelly et al., 1999; Cosgrove, 2000, 2001; De Veylder et al., 2001; Breuninger and Lenhard, 2010; Wolf et al., 2012; Kalve et al., 2014; Szymanski, 2014). Though overlapping, these two processes are separated in time, allowing kinematic growth analysis to calculate average rates of cell division and expansion. Such studies provide insights into the variation in cellular processes and help to understand how the activity of individual cells defines the fate of a mature leaf (Silk and Erickson, 1979; De Veylder et al., 2001).

The plant growth hormone auxin regulates leaf growth and development by controlling leaf positioning, initiation, differentiation and venation patterning (Reinhardt et al., 2000, 2003; Benkova et al., 2003; Scarpella et al., 2006). Local auxin accumulation mediated by PIN-FORMED1 (PIN1) efflux proteins specifies the site of leaf primordium initiation, while its depletion from the proximity inhibits the formation of additional primordia (Reinhardt et al., 2003; Heisler et al., 2005; Vernoux et al., 2010). Basipetal transport of auxin through the subepidermal layers of a developing primordium leads to the formation of a midvein and lateral veins (Scarpella et al., 2006). Auxin also influences formation of leaf serrations by generating PIN1 localization-driven auxin maxima in the lobes at the leaf margin (Hay et al., 2006; Bilsborough et al., 2011). In brief, auxin transport not only plays a pivotal role in early leaf initiation, but also in the later phases sculpting mature leaf shape and form.

Directional flow of auxin occurs by active polar transport with the help of AUXIN1/LIKE AUX1 (AUX/LAX) influx carriers and efflux carriers such as PINs and ABC transporters (Křečk et al., 2009; Kang et al., 2011; Swarup and Péret, 2012). Disruption of this auxin transport causes defects in many developmental and growth-related processes (Friml, 2003). PINOID (PID) is an early auxin-inducible gene belonging to the AGC VIII group of protein-serine/threonine kinases (Robert and Offringa, 2008) that plays a major role in the control of polar auxin transport (PAT; Bennett, 1995; Christensen et al., 2000; Benjamins et al., 2001). PID plays a controlling role in PINs’ subcellular distribution, since changes in PIN1, PIN2, and PIN4 localization in the plasma membrane switches from the basal to the apical side of the cell when PID abundance passes above a certain threshold (Friml et al., 2004).

Here, we show the effect of altered *PID* expression on auxin and we study the effect of these changes on Arabidopsis leaf growth. While *pid* knockouts had no significant changes in total auxin levels, *PID^OE^* contrastingly, accumulated auxin in the leaves. Previous reports showed that allelic mutants of *pid* show pleiotropic growth defects (Bennett, 1995) and *PID^OE^* lines display an agravitropic root phenotype and reduced number of lateral roots (Benjamins et al., 2001). However, leaf phenotyping, especially with cellular resolution, has not received much attention. In our study, *PID^OE^* lines showed a strikingly reduced rosette growth, encouraging us to study the cellular and genetic basis of this interesting phenotype.

## Materials and Methods

### Plant Material and Growth Conditions

Seedlings of *Arabidopsis thaliana* Col-0 ecotype were grown in half strength Murashige and Skoog (MS) medium including vitamins (Duchefa, The Netherlands), containing 1% sugar, 0.5 g/L MES buffer and 0.7% agar at 21°C with a light intensity of 70–90 μmol m^-2^s^-1^ in long day conditions. Prior to sowing, seeds were sterilized with 70% ethanol for 30 s, subsequently with 5% bleach and sterile water. Knockout T-DNA insertion lines were obtained from NASC. The *pid-14* mutant was the SALK_049736 line as reported by Haga et al. (2014). The two *PID^OE^* lines, P10 and P21, were developed by Benjamins et al. (2001) by cloning the *PID* cDNA in sense orientation behind the strong Cauliflower Mosaic Virus 35S promoter (35S::PID) and introducing it into *A. thaliana* ecotype Columbia (Col).

### Microscopic Morphological Analysis and Kinematic Growth Analysis

Rosette pictures were taken from three replicate plates using a Cannon EOS 40D camera. Leaves were cleared overnight with 70% ethanol, followed by 100% lactic acid. Cleared leaves were then pictured under a Nikon AZ-100 macroscope equipped with a Nikon DS-Ri1 digital camera. Pictures of young leaves and epidermal cells were taken with a Nikon C1 confocal microscope (Nikon, Belgium) using 20–60× lenses depending on the age. Three leaves, with an area close to the average, were chosen for drawing cells in ImageJ^1^. The drawn cell pictures were saved as eight bit and 2556 × 2045 size, in jpeg format and later analyzed in a linux-based software and used for further calculations as in Andriankaja et al. (2012). Cell measurements for the petiole were made by staining petioles in propidium iodide as mentioned in Wuyts et al. (2010) followed by visualization using a Nikon C1 confocal microscope.

### Transverse Sectioning

In brief, fixed leaves were dehydrated by sequential incubation in different concentrations of ethanol and embedded in Technovit 7100 resin (Kalve et al., 2015). Using a rotary microtome, 5 μm thick sections of the middle part of leaves were made, stained and mounted on slides before visualization.

### GUS Staining

Whole plants were cleared in acetone for 10 min followed by a 10 min treatment with GUS solution (Phosphate buffer (pH 7), 0.1% Triton X-100, 0.5 mM K_4_[Fe(CN)_6_].3H_2_0, 0.5 mM K_3_[Fe(CN)_6_]) without *X*-gluc before being kept at 37°C in GUS staining solution with *X*-gluc (958 μM *X*-gluc dissolved in 1% DMSO). Samples were fixed with ethanol:acetic acid (3:1) after 3–5 h and cleared with 8 M NaOH before mounting on slides.

### Auxin Quantification

To measure concentrations of free and conjugated IAA, leaves 1 and 2 were harvested every day from 9 to 25 DAS. The analyses were repeated twice as independent experiments and from multiple plates in each experiment. Dissected samples were collected, frozen in liquid nitrogen, and ground using a MagNALyser (Roche) with 2 mm glass beads. IAA was extracted based on Prinsen et al. (2000). Homogenized leaves were extracted overnight in 80% methanol (10 ml/g fresh weight). C_6_^13^-phenyl-IAA (150 pmol, Cambridge Isotope Laboratories Inc., Andover, MA, United States) was added as internal standard. After centrifugation (20000 *g*, 15 min, 4°C, 5810R, rotor FA-45-30-11 Eppendorf, Hamburg, Germany), part of the supernatant was passed over a C18 cartridge (500 mg, Varian, Middelburg, The Netherlands) to retain pigments. The effluent was then diluted to 50% methanol and concentrated on a DEAE-Sephadex (2 ml, GE Healthcare Bio-Sciences AB, Uppsala, Sweden) anion exchange column for the analysis of free IAA. The DEAE cartridge was eluted with 10 ml 6% formic acid and IAA was concentrated on a C18 cartridge coupled underneath. This C18 cartridge was then eluted with 2 × 0.5 ml diethylether and the ether was evaporated (Turbovap), dissolved in acidified methanol and methylated with diazomethane. The samples were subsequently dried under a nitrogen stream and dissolved in 50 μl 10% MeOH. For small biomasses, samples were diluted in 6% formic acid and immediately concentrated on a C18 cartridge omitting the DEAE anion exchange cartridge. The remaining part of the supernatant was diluted ½ with 14 N NaOH and hydrolysed at 100°C for 3 h under a water saturated nitrogen stream. After hydrolysis, samples were titrated to pH-3 with 1 M HCl, diluted 1/10 with water and concentrated on a C18 cartridge as described above for free IAA (Prinsen et al., 2000).

Indole-3-acetic acid was analyzed by UPLC-MS/MS (Acquity TQD, Waters, Manchester, United Kingdom) (6 μl injection by partial loop, column temp. 30°C, solvent gradient 0–2 min: 95/5; 10% MeOH in NH4OAc 1 mM/MeOH; 2–4 min linear gradient until 10/90 10% MeOH in NH4OAc, 1 mM/MeOH; 4–6 min, isocratic 10/90 10% MeOH in NH4OAc 1 mM/MeOH; MS conditions: Polarity MS ES(++), capillary 2 kV, cone 20 V, collision energy: 20 eV, source temperature: 120°C, desolvation Temperature: 450°C, Cone gas flow 50l/h, desolvation gas flow: 750 l/h, collision gas flow: 0.19 ml/h). For quantification we selected the diagnostic transitions 190 > 130 m/z for Me-IAA and 196 > 136 m/z for Me-C^13^-IAA (dwell time 0.020 s) using Masslynx and Quanlynx software (V4.1, Waters). Methanol and water used for MS were UPLC grade (Biosolve, Valkenswaard, The Netherlands). Data are expressed in pmol per gram fresh weight (pmol.g^-1^FW).

### Flow Cytometry

Leaves were harvested as for auxin quantification. Prior to analysis the frozen leaves (kept on dry ice) were chopped with a single edge razor blade in 200 μl Crystain UV precise P Nuclei extraction buffer (Partec) and 800 μl Cystain fluorescent buffer (Partec). The mix was filtered through a 50 μm CellTrics filter and transferred to a glass tube. At least 10,000 nuclei were analyzed with a CyFlow flow cytometer and the FloMax software (Partec) in six independent biological replicates for each genotype. Endoreduplication index was calculated as

$$\begin{array}{c} {EI=0}^{*}{2C+1}^{*}{4C+2}^{*}{8C+3}^{*}16C. \end{array}$$

### Confocal Imaging

Seedlings of DR5_rev_::GFP and crossed with *PID^OE^* lines were stained with propidium iodide (0.1 mg ml^-1^) for visualization of cell walls. A Nikon C1 confocal microscope (Nikon, Brussels, Belgium) was used for GFP visualization.

### Expression Profiling

For expression analysis RNA was isolated using Purelink Plant RNA reagent (Ambion Life Technologies) and quantified with a nanodrop NZ 1000 (Thermo scientific). An average of 1 μg of RNA was used for first strand cDNA synthesis using RQ1 RNase-Free DNase treatment and the GoScript^TM^ Reverse Transcription System (Promega). A PCR of 30 cycles, using *ACT 8* primers and gene specific primers spanning the intron region, was performed with 54°C as annealing temperature (primers in Supplementary Table 1). The time course SyBr green assay for qPCR was accomplished as per the developer’s protocol using ROX SYBR Mastermix blue dTTP (Takyon) and a Step one plus thermocycler (Life technologies). This was done as three biological and technical repetitions using *ACT8* as the reference gene. The results were analyzed as ΔΔCT comparison with the StepOnePlus^TM^ Real-Time PCR System (Life Technologies^TM^) software with a confidence level set at 95%.

### RNA Sequencing

Eighteen RNA samples, originating from the first pair of leaves at 9 and 16 DAS and from WT and *PID^OE^* lines were commercially sequenced using the Illumina^TM^ platform. Prior to library preparation the RNA quality and integrity was assessed per Illumina^TM^ guidelines. Library preparation was done using the TruSeq^®^ Stranded mRNA sample preparation 96-reaction kit (Illumina^TM^) following the low sample protocol according to manufacturer’s recommendations. In brief, approximately 2.5 μg of total RNA was diluted and purified using RNA purification beads targeting the poly-A tail of the mRNA and this was subsequently fragmented by means of the enzymes provided in the kit. After the cDNA synthesis adenylation of 3′ ends and ligation of the adaptors were performed. Adaptors were ligated in 12-plex formations, allowing the pooling of 12 samples. Subsequently, the library was quantified using PicoGreen^®^ dye (Life Technologies^TM^) as described in the manufacturer’s protocol. To accurately quantify the concentration in nM of the sample, the Kapa SYBR^®^ FAST universal qPCR kit (Kapa Biosystems^TM^) for Illumina^TM^ sequencing was used to quantify the number of the amplifiable molecules in the library and the Bioanalyzer^®^ machine (Agilent Technologies^TM^) to determine the average fragment size. These measurements allowed optimizing the flow cell clustering prior to the Run. The library was 50 bp pair-end sequenced in one lane of an Illumina^TM^ HiSeq1500 sequencer.

### Data Analysis

Resulting sequence data was preliminary analyzed by CLC Genomics Workbench v.6 using *Arabidopsis thaliana* (Col-0 TAIR10) sequence database^2^ as reference genome. The RNA-Seq analysis was carried out for sequence reads obtained from the three genotypes. Throughout the analysis with CLC default settings were used. Briefly, after the trimming of the sequences they were mapped against the reference genome with the default settings. The expression values were calculated based on “reads per kilo base of exon model per million mapped reads” (RPKM) values (Mortazavi et al., 2008). The RNA-seq data was grouped accordingly and two group comparisons (unpaired) were performed. The expression values were normalized by scaling to the default setting of 10 million reads, and the proportions were compared using Baggerley’s test (Baggerly et al., 2003). All significantly induced or repressed genes with known functions were classified into groups based on gene ontology (GO) information obtained from the TAIR Database^2^ by using MapMan (Thimm et al., 2004) and overrepresented functions and gene enrichment studies were carried out by PageMan (Usadel et al., 2006) and Cytoscape (using the BiNGO plugin) software.

### Statistics

All the measurements were analyzed by *t*-test (*P* < 0.05) using the R statistical package^3^. Conditions of normality of distribution and homogeneity of variance were checked and met.

### Accession Number

The Arabidopsis Genome Initiative locus identifier for the *PID* gene is AT2G34650.

## Results

### Spatiotemporal Expression Pattern of *PID*

**FIGURE 1:**
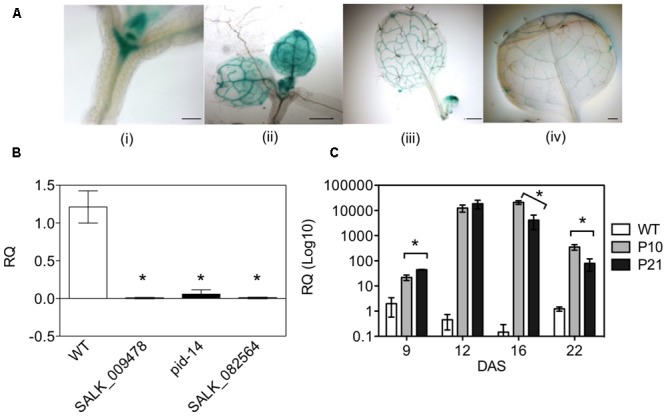
Spatio-temporal expression of *PID* in Arabidopsis plants. pPID::GUS line with expression in shoot apical meristem and the proliferating leaves at 9 DAS (i, ii); and at 14 and 17 DAS in the expanding leaves (iii, iv) **(A)**. Expression profiling of T-DNA insertion mutants and overexpression lines of *PID*. SyBr green qPCR assay using the seedlings of *pid* mutants at 7 DAS **(B)** and leaves of *PID* overexpression lines at different time points **(C)**. Asterisks mark the difference between WT and mutants **(B)**. Data are averages ± SE (*t*-test, *P* < 0.05); *n* = 3; Scale bar 100 μm (i) and 500 μm (iii, ii, iv).

To better understand the role of PID-directed auxin transport in leaf development, we first studied *PID*-expression during the development of the Arabidopsis leaf. p*PID::GUS* lines showed high levels of *PID* expression in the shoot apical meristem and in the newly formed primordia. Later during leaf development, expression became restricted to the vasculature of expanding leaves (**Figure 1A**). The spatial and temporal activity of the *PID* promoter pointed toward a stage-specific role in leaf development.

### Characterization of *PID* Mutants

Semi-quantitative RT-PCR at 9 DAS (days after stratification) on the leaves of wild type (WT) and two *PID* overexpression (*PID^OE^*) lines, P10 and P21, showed a clear increase in *PID* expression in both lines (Supplementary Figure 1). qPCR analysis on single *pid* mutants (*pid-14*, SALK_082564, SALK_009478) detected a decreased to nearly absent *PID* expression in the seedlings at 7 DAS (**Figure 1B**). A time series of qPCR analyses confirmed the overexpression in both *PID^OE^* lines and revealed that in early growth stages (9 and 12 DAS) overexpression was highest in P21. Interestingly, in expanding and mature tissues (16 and 22 DAS) *PID* transcript level was highest in P10 (**Figure 1C**).

### IAA Measurements

**FIGURE 2:**
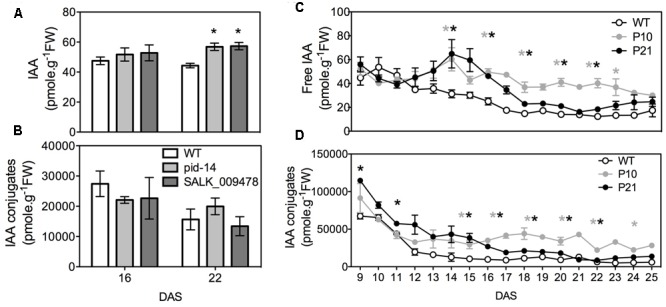
Indole-3-acetic acid (IAA) measurements. Analysis of free **(A)** and conjugated IAA **(B)** in wild type and *pid* mutants. Analysis of free **(C)** and conjugated IAA **(D)** in *PID^OE^*, P10 and P21 from proliferating, expanding and mature first pair of leaves. Error bars represent averages ± SE (*t*-test, *P* < 0.05). Asterisks mark significant differences between different lines and the wild type (gray: WT vs. P10, black: WT vs. P21 in **C,D**).

Since *PID* is an auxin transport regulator we measured IAA (indole-3-acetic acid) concentrations throughout the development of the first pair of leaves from 9 to 25 DAS in *PID^OE^* lines and at two time points in *pid* knockout lines (due to comparatively weaker phenotypes; see later). *pid* knockouts had no significant difference in free or conjugated IAA levels in the leaves compared to the WT at 16 DAS, while both knockouts (*pid-14* and SALK_009478) showed elevated free IAA levels at 22 DAS. However, the total IAA pool (free IAA + conjugates) remained unchanged compared to the WT (**Figures 2A,B**). *PID* overexpression, on the other hand, led to increased free IAA and IAA-conjugate levels in the leaves. Over time, free IAA levels dropped in the WT, whereas this was not obvious in both *PID^OE^* lines (**Figure 2C**). After D16, the free IAA levels also started to drop in the *PID* lines, in P21 almost to WT levels, whereas they remained significantly higher in P10. In the early days, P21 had comparatively higher IAA-conjugate levels and interestingly, later on, it was intermediate to P10 and WT (**Figure 2D**).

### Auxin Response and Transport in the Leaves of WT and *PID* Overexpression Lines

**FIGURE 3:**
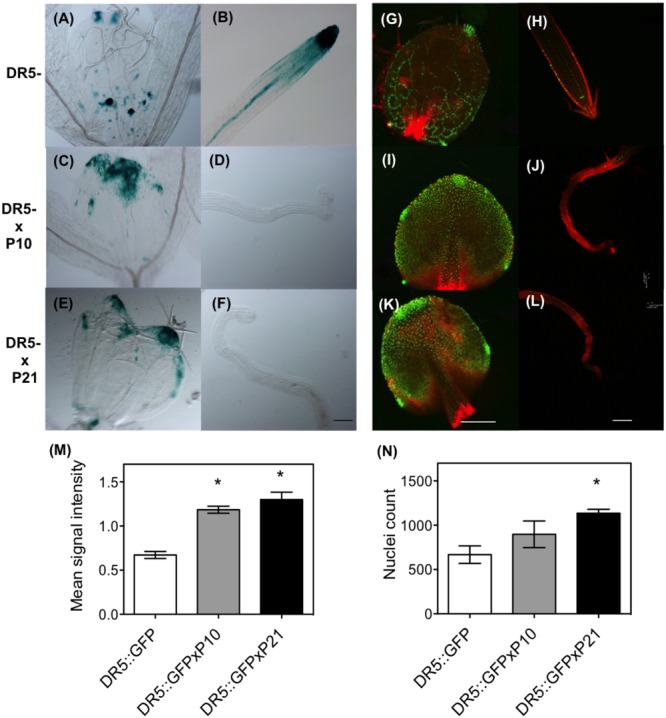
Effect of *PID* overexpression on auxin signaling in Arabidopsis. GUS assay with DR5::GUS **(A,B)**; DR5::GUS in *PID^OE^* P10 **(C,D)** and P21 background **(E,F)** at 9 DAS in the leaves and roots. Confocal images with DR5_rev_::GFP signal in control **(G,H)** and DR5_rev_::GFP × *PID^OE^* P10 **(I,J)** and DR5_rev_::GFP × *PID^OE^* P21 **(K,L)**. Measurements of intensity of GFP signal **(M)** and count of nuclei expressing GFP using the leaves from various mutant lines **(N)**. Asterisks indicate significant differences between the transgenic lines and the wild type. Error bars represent ± SE (*t*-test, *P* < 0.05). Scale bar 100 μm for GUS assay and 50 μm for confocal images.

Since *PID* overexpression has a positive effect on auxin levels, this could consequently alter auxin signaling across the leaf. To investigate the effects of *PID^OE^* on auxin signaling we compared the DR5::GUS and DR5_rev_::GFP sensors in the wild type and *PID* overexpression backgrounds. Analysis of the leaves clearly showed that *PID^OE^* resulted in a more pronounced accumulation of GUS and GFP signal in the top of the leaf blade and around the leaf margins, compared to WT plants (**Figures 3A,C,E,G,I,K**). In addition, the signal was clearly visible in the root apex of the WT, but was lower to nearly absent in the collapsed roots of both P10 and P21 lines (**Figures 3B,D,F,H,J,L**). Quantification of signal intensity from the leaves of DR5_rev_::GFP lines proved the increased GFP signal in the *PID* overexpression background (**Figures 3M,N**).

### Pleiotropic Growth Defects of *PID* Overexpression Lines and *PID* Mutants

**FIGURE 4:**
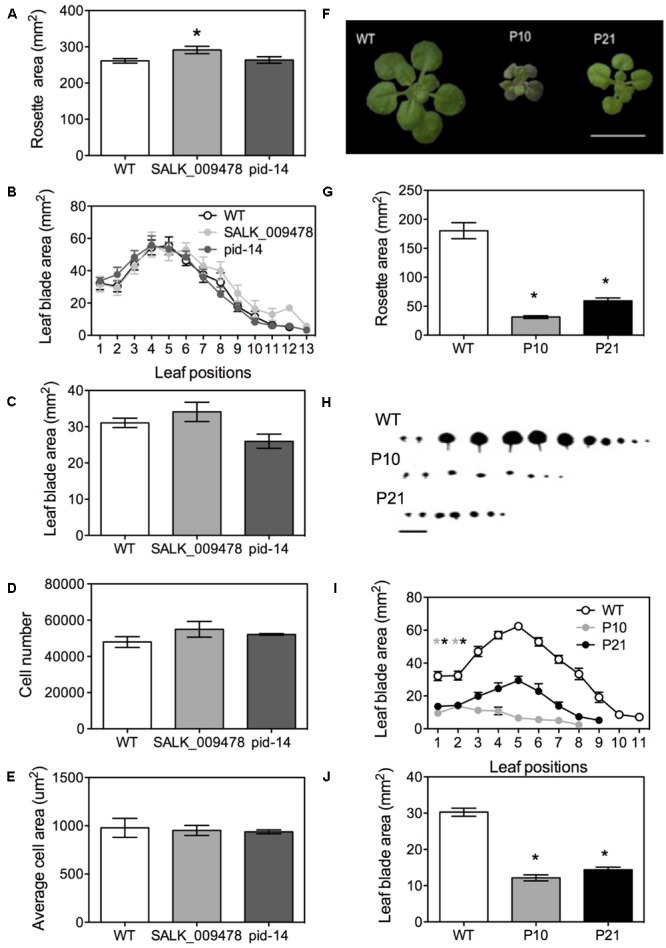
Phenotypes of *PID* overexpression lines and *pid* mutants in Arabidopsis. Rosette area of *pid* knockouts at 22 DAS **(A)**. Leaf series at 25 DAS **(B)**. Leaf blade area in *pid* knockouts **(C)**. Cell number **(D)** and average cell area **(E)** in first pair of leaves in *pid* knockouts. Figure showing mature rosettes at 22 DAS in WT and *PID* overexpression lines **(F)**. Rosette area measurements at 22 DAS **(G)**. Leaf series **(H)** and leaf series measurements at 25 DAS starting from oldest (first L1, L2) to youngest leaves (L9-L11; **I**). Leaf area measurements for the first leaf pair **(J)**. Asterisks indicate significant differences with the wild type. Error bars represent ± SE (*t*-test, *P* < 0.05). Scale bar = 10 mm.

Rosettes and leaves of knockouts showed distinct morphological changes (**Figures 4A–C**) including a slightly bigger rosette area (15% in SALK_009478; **Figure 4A** and Supplementary Figure 2A) and the presence of one or two additional leaves compared to wild type as previously reported by Bennett (1995) in the allelic *pid* mutant lines (**Figure 4B**). The blade areas of the first pair of leaves of the knockout mutants were similar to the WT (**Figure 4C**). Cellular image analysis showed no significant differences between WT and the knockouts in terms of cell number per leaf and average cell area (**Figures 4D,E**). *pid-14*, SALK_082564 and SALK_009478 occasionally showed three cotyledons (Supplementary Figure 2B). In the mutants with three cotyledons, the first leaf pair developed alternate to cotyledons instead of opposite as in the normal phyllotaxy (Supplementary Figure 2C). Homozygous lines of *pid-14* (Bennett, 1995; Haga et al., 2014) and SALK_082564 were sterile and had a *pin*-like inflorescence (Supplementary Figures 2D,E). However, as leaf growth was not drastically affected in these knockout lines, they were not included in the subsequent detailed leaf development studies.

In contrast, both *PID^OE^* lines, P10 and P21, had significantly smaller rosettes (82 and 67%, respectively) than the WT at 22 DAS, (**Figures 4F,G**). Leaf series of the three lines also indicated that fewer leaves were formed on the individual rosettes, 10 ± 2 in WT and 6 ± 1 in *PID^OE^* lines (**Figures 4H,I**). In addition, using leaves 1 and 2, which are indistinguishable in their morphology and have synchronized growth, we showed that P10 and P21 had 60 and 52% smaller mature leaf blade areas than the WT (**Figure 4J**). Petioles were small in *PID^OE^* lines, mainly due to fewer cells and not to their sizes (Supplementary Figures 3A–C). Transverse sections of leaves in the expansion phase revealed that increased *PID* levels not only affected leaf growth in the horizontal plane of the blade, but also in the dorsoventral direction. Transverse sections showed a clear increase in thickness of the leaf in P10 and a slighter increase in P21, compared to the WT (Supplementary Figures 3D–F). While the number of layers remained unchanged, spongy palisade cells were clearly enlarged in the dorsoventral direction (Supplementary Figures 3G,H). Consistent with earlier observations (Kleine-Vehn et al., 2009) both overexpression lines showed a thick vasculature system (Supplementary Figures 3I–L).

### Kinematic Growth Analysis

**FIGURE 5:**
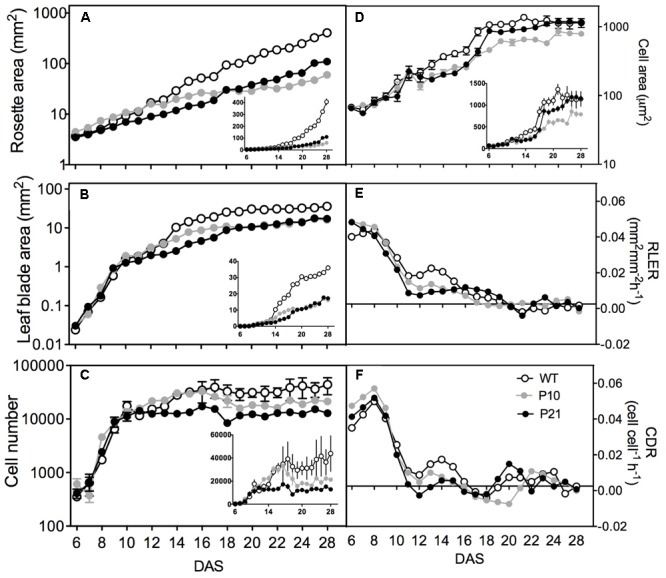
Kinematic growth analysis of the first leaf pair of Arabidopsis wild type and *PID* overexpression lines. Rosette area, *n* = 40–50 **(A)**. Leaf blade area, *n* = 24–26 **(B)**. Average cell number in a leaf, *n* = 6 **(C)**. Average cell area, *n* = 6 **(D)**. Relative leaf expansion rate **(E)**. Cell division rates **(F)**. Insets show the same on a linear scale. Error bars represent averages ± SE of two replicate experiments.

To understand the cellular basis of the leaf phenotype due to the altered auxin levels, we performed a kinematic analysis of the first leaf pair. From 6 to 28 DAS, we measured rosette area of WT and the two *PID^OE^* lines from approximately 100 seedlings (performed in duplicates). P10 had a 20% larger rosette than WT in the early growth phases (**Figure 5A**), most likely due to its bigger seed size (Supplementary Figure 4). At 28 DAS, however, P10 (60.0 ± 5.5 mm^2^) and P21 (110.1 ± 6.5 mm^2^) rosettes were smaller than those of the WT (406.8 ± 25.7 mm^2^). From the same seedlings that were used for the rosette measurements, six average sized leaves were harvested daily for measurement of leaf blade area (**Figure 5B**) and calculation of leaf expansion rate (**Figure 5E**). At 28 DAS, there was a 55 and 52% reduction in the size of mature leaves in P10 and P21 compared to the WT, respectively. Differences in leaf expansion rates were most prominent between 10 and 15 DAS, where the WT exhibits higher expansion rates than both *PID^OE^* lines, resulting in the larger leaf area (cf. **Figures 5B,E**).

The detailed cellular analysis showed that during the early stages of leaf development, cell proliferation was fairly similar in the three lines. The differences lay in the duration of cell formation, as P21 stopped to proliferate from 10 DAS onward (**Figure 5C**), cell formation in P10 was arrested around 12 DAS, whereas the WT still produced new cells until 15 DAS, resulting in more pavement cells in mature WT leaves compared to the two *PID^OE^* lines. This difference was also reflected in the cell division rate where between 10 and 17 DAS the WT still exhibited a significant division rate, whereas the rates in the *PID^OE^* lines were close to zero in this period (**Figure 5F**). Surprisingly we observed an overall reduction in the stomatal index, i.e., the ratio of guard cells and the total number of epidermal cells, in the *PID^OE^* lines, suggesting an affected meristemoid cell division process (Supplementary Figure 5). From 12 DAS on, cell size of *PID^OE^* lines lagged behind those of the wild type. The difference in cell area between WT and P10 extended to the mature phase. The pavement cell area of P21 leaves, however, catched up from 16 to 17 DAS onward (**Figure 5D**).

**FIGURE 6:**
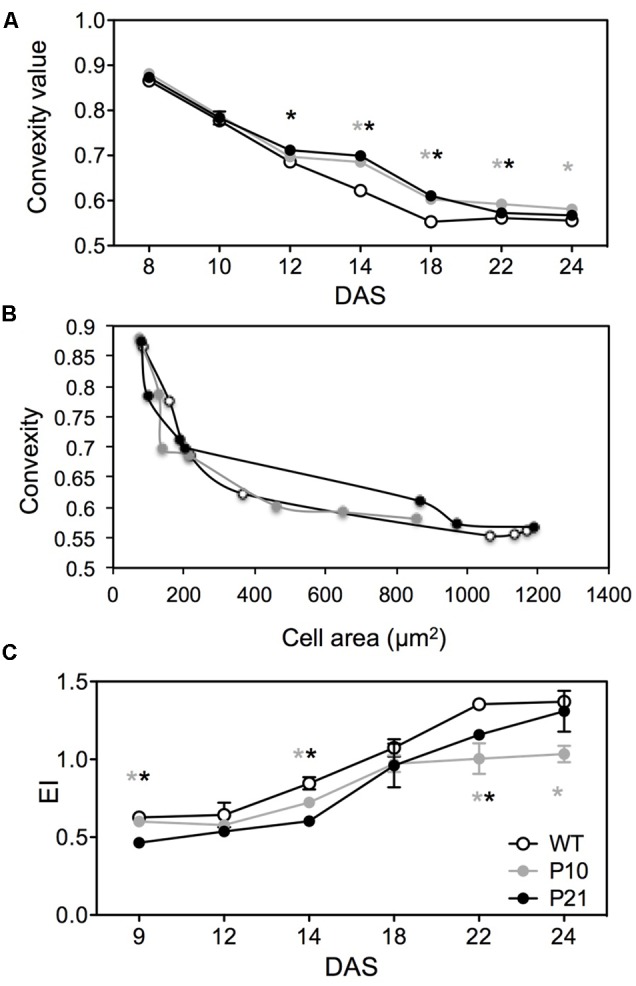
Cell shape and endoreduplication in wild type and *PID* overexpression lines. Comparison of pavement cell convexity in the abaxial epidermal layer at different time points between WT, P10 and P21 **(A)**. Convexity and cell area correlation between different genotypes over time **(B)**. Comparison of ploidy status (EI = 0^∗^2C+1^∗^4C+2^∗^8C+3^∗^16C) of various lines by means of number of endocycles undergone by nuclei **(C)**. Error bar ± SE (*t*-test, *P* < 0.05). Gray and black asterisks represent significant difference of P10 and P21 compared to the WT, respectively.

In addition, the convexity of epidermal pavement cells differed between the WT and the two *PID^OE^* lines between 12 and 22 DAS (**Figure 6A**). As defined by Hectors et al. (2010) convexity is the cell area in relation to the area of its convex hull. More complex cell shapes have lower convexity values and it approaches 1 for spherical cells. We used CellP software for calculating cell convexity for the jigsaw-shaped adaxial pavement cells. Epidermal cells in *PID^OE^* leaves were less complex, but this could be associated with a smaller average size rather than the effect of *PID* overexpression on cell shape differentiation. Indeed, the relationship between cell area and convexity appears to be unaffected in both *PID^OE^* lines (**Figure 6B**).

### Endoreduplication Index

In Arabidopsis, the size of pavement cells shows a positive correlation with DNA-ploidy levels, determined by the number of endocycles (successive rounds of DNA replication in the absence of mitosis) they have undergone (Melaragno et al., 1993). Therefore, we measured ploidy levels and the number of endoreduplication cycles using flow cytometry at different time points during development. Consistently, the endoreduplication index was reduced in *PID^OE^* lines compared to WT throughout development (**Figure 6C**). In contrast, in P21, ploidy levels were initially lowest, but increased to wild type levels from the expanding stage onward, paralleling the development of cell size (**Figure 5D**) and reflecting the *PID* expression and IAA levels in P21 at that stage (**Figures 1C, 2C,D**).

### Transcriptome Analysis by RNA Sequencing

**FIGURE 7:**
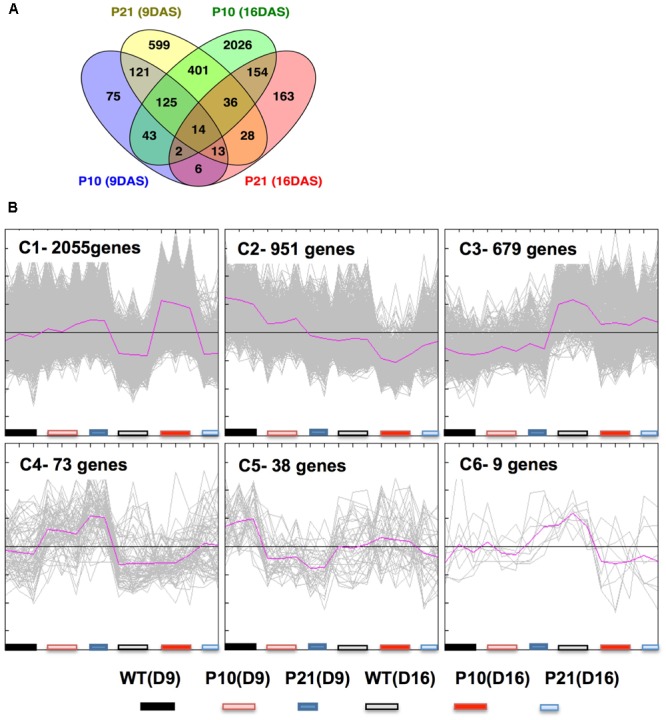
Transcriptome analysis of wild type and *PID* overexpression lines at 9 and 16 DAS. Overlap between differentially expressed genes as a result of *PID* overexpression in P10 and P21 at two time points as shown by Venn diagram **(A)**. Clustering of gene expression patterns by QT-Cluster analysis of significantly changed genes. The number of genes in each cluster and cluster numbers are mentioned at the upper left corner **(B)**.

To understand the transcriptional changes induced by increased auxin levels that led to reduced cell division and expansion, we performed RNA sequencing on proliferating (9 DAS) and expanding (16 DAS) leaves of *PID^OE^* and WT (Supplementary Data 1). Collectively, 3805 genes were differentially expressed at least in one condition using FDR-corrected *p*-value < 0.05 and log_2_ fold change > 0.75, between *PID^OE^* and WT (Supplementary Data 2). There was only little overlap in number of genes differentially expressed between P10 and P21 (**Figure 7A**). Clustering of differentially expressed genes was done by QT-Clust analysis (Pearson correlation; cluster diameter-0.8, minimum cluster size-15). Clusters 1, 2, 4, 5, and 6 show that the effect of P21 was strongest in proliferating tissues (at 9 DAS) and Clusters 1 and 2 reflect the prominent effect of P10 in the expanding tissues (at 16 DAS) (**Figure 7B**). These patterns therefore closely correlate with *PID* expression (**Figure 1C**) and IAA accumulation (**Figures 2C,D**) at these stages.

**FIGURE 8:**
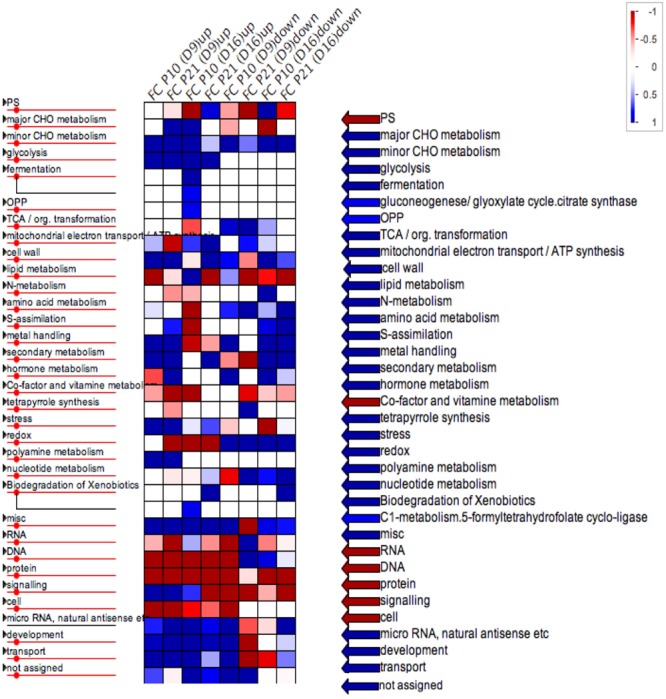
PageMan representation of differentially expressed genes in two *PID* overexpression lines. Overrepresented biological pathways in *PID^OE^* lines at different time points are highlighted in blue. Upregulation and downregulation is as indicated in the legend above.

PageMan gene enrichment analysis for significantly induced and repressed genes showed that upregulated biological categories included carbohydrate metabolism and glycolysis, photorespiration, cell wall modifications (cellulose and hemicellulose synthesis etc.), secondary metabolite synthesis, biotic-abiotic stress, signaling and transport. Pathways that were enriched among downregulated genes included the TCA cycle, redox, nucleotide and amino acid metabolism (**Figure 8**). In general, hormone metabolism-related genes indicated upregulation of jasmonic acid, gibberellins and ABA metabolism (Supplementary Data 2; Saini et al., submitted results).

### Auxin Related Transcriptional Changes in the *PID* Overexpression Lines

**FIGURE 9:**
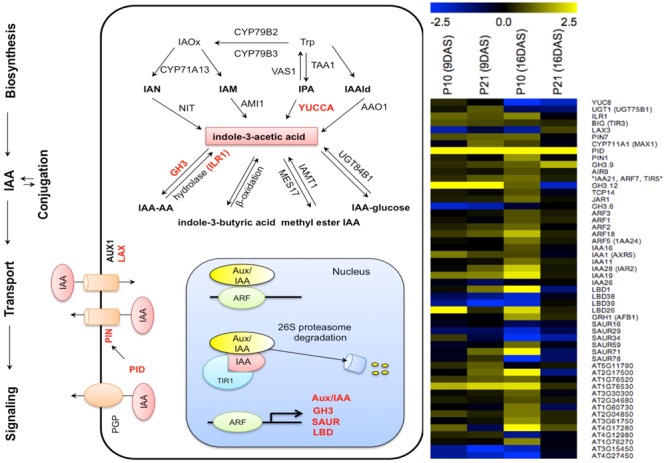
Effect of *PID* overexpression on the expression of genes involved in various auxin-related pathways. Schematic representation of auxin related pathways, on which affected genes are indicated in red, based on RNA-seq data from both *PID^OE^* lines. Heat map shows auxin-related genes affected by *PID* overexpression. Abbreviations: Trp, tryptophan; IAOx, indole-3-acetaldoxime; IAN, indole-3-acetonitrile; IPA, indole-3-pyruvate; IAM, indole-3-acetamide; IAAld, indole-3-acetaldehyde. Yellow and blue colors in the heat map represent up- and downregulation of genes, respectively.

To understand the reason for high auxin levels and response in *PID^OE^* lines, we studied the transcriptional changes of genes related to auxin homeostasis and signaling in more detail using the first leaf pair. None of the auxin biosynthesis genes (based on the review, Woodward and Bartel, 2005) showed changes in their expression levels except *YUC8*, which was downregulated at 16 DAS in both *PID^OE^* lines, whereas genes related to conjugation pathways varied over time and between the lines (**Figure 9**). An IAA deconjugating gene, *IAA-LEUCINE CONJUGATE HYDROLASE* (*ILR1*), showed upregulation in P10 at 16 DAS. The auxin-inducible *GRETCHEN HAGEN3* (*GH3*) class of genes encodes IAA-amido synthetases, which convert excess free IAA to IAA-amino acid conjugates, and therefore controls endogenous auxin levels (Staswick et al., 2005). Downregulation of *GH3.6* at 9 DAS and upregulation of *GH3.9* at 16 DAS (∼2-fold in P10 and ∼4-fold in P21) indicated that the shift between deconjugation and conjugation between the two time points could have contributed to the gradually increasing and then decreasing levels of free IAA. Also, *INDOLE-3-BUTYRIC ACID RESPONSE* (*IBR1* and *IBR3*) genes, required for conversion of IBA to IAA (Korasick et al., 2013), were upregulated in P10 at 16 DAS. The differences in upregulation of conjugation and deconjugation genes between the two lines could account for differences in their IAA pool in later stages, as shown in **Figures 2C,D**.

Among the genes related to polar auxin transport, *PIN1* showed a threefold increase in P10, *PIN7* showed a twofold increase in P21 at 9 DAS and in P10 at 16 DAS, whereas the influx protein *LAX3* showed a threefold decrease in P10. This could be interpreted as a potential change in influx and efflux of auxin from cells in *PID^OE^* leaves.

*AUXIN/INDOLE-3-ACETIC ACID (Aux/IAA), GH3, SMALL AUXIN UP RNAs (SAUR), LATERAL ORGAN BOUNDARIES-DOMAIN (LBD)* are known as primary auxin responsive genes and are directly regulated by Auxin Responsive Factors (ARFs; Okushima et al., 2007; Paponov et al., 2008, 2009; Lee et al., 2009). All the significantly changed ARFs (*ARF1, ARF2, ARF3, ARF18*, and *ARF5*) and IAAs (*IAA1, IAA11, IAA16, IAA19, IAA24, IAA28*, except *IAA26*) showed upregulation at different time points in at least one of the genotypes. *LBD1* and *LBD26* showed a strong upregulation, while *LBD38* and *LBD39* showed strong downregulation in both *PID^OE^* lines (**Figure 9**). The general upregulation of auxin response genes correlates with the elevated auxin levels and observed increased DR5 activity (**Figures 2, 3**).

### Transcriptional Changes in Cell Division and Expansion-Related Genes

To understand the effect of PID-induced auxin accumulation on cell division and expansion at the transcriptional level, changes in the expression levels of core cell cycle and cell wall-related genes were investigated. The RNA sequencing data showed modulations of many core cell cycle genes (based on Vandepoele et al., 2002; De Almeida Engler et al., 2009), including the downregulation of major cyclins (*CYCA2;4, CYCB2;2, CYCD5;1*, and *CYCH;1*), Cyclin Dependent Kinase subunit 2 (*CKS2*), *ANAPHASE-PROMOTING COMPLEX (APC10* and *APC8)*, the E2F pathway genes, and *KIP RELATED PROTEIN KRP3* and *KRP7*, with the exception of upregulation of *KRP1* and *KRP2* (Supplementary Table 2). Together these results suggest that PID-induced alterations in auxin levels could have affected the cell cycle through these genes, which could be interpreted as the reason for suppressed proliferation during development.

In plants, the extent of cell expansion largely depends on the biomechanical properties of their cell walls. The expression levels of numerous cell wall-related genes (based on Mapman notation; bin 11; Thimm et al., 2004) were affected in the same sense in both *PID^OE^* lines, though with a different magnitude (Supplementary Table 3). Genes related to cellulose and hemicellulose were upregulated in both lines. One of the cellulose synthase genes involved in secondary wall formation, *CesA4* (*IRX5*) (Taylor et al., 2003), was upregulated in P10 at 16 DAS. Some cellulose synthase-like gene family (Csl) were also among upregulated genes such as *CslA, CslC, CslE*, and *CslG* family members in P21 at 9 DAS and P10 at 16 DAS. Dynamic changes in cell area of *PID^OE^* lines, especially in P21 after 16 DAS, and the thicker leaves in P10 could also be due to the changes in the cell wall modification proteins such as xyloglucan endotransglucosylase/hydrolases (XTHs) and expansins (Cosgrove, 2000; Nishitani and Vissenberg, 2007; Van Sandt et al., 2007). Indeed, changes in alpha and beta expansins and XTHs were observed in our transcriptome data (Supplementary Table 3).

### Transcriptional Regulation of Leaf Development in *PID* Overexpression Lines

According to a proposed model, MONOPTEROS (MP) and PIN regulate vein formation under the influence of auxin, where MP acts upstream of PIN and could also be regulating *PIN* expression directly or indirectly (Wenzel et al., 2007). MP and NONPHOTOTROPIC HYPOCOTYL 4 (NPH4) work synergistically in development of the midvein and differentiation of secondary and tertiary veins. HOMEOBOX GENE 8 (ATHB8) controls preprocambial development and procambium differentiation required for vascularization (Hardtke et al., 2004; Wenzel et al., 2007; Donner et al., 2010). Both *PID^OE^* lines, and especially P10, showed a thick vasculature (Supplementary Figure 3) that could be explained by the upregulation of *PIN1, MP, NPH4*, and *ATHB8* at 16 DAS. Transcription factors KANADI, YABBY family members, and ETTIN/ARF3, are required for the identity of the abaxial domain of the leaf (Pekker et al., 2005; Stahle et al., 2009; Bonaccorso et al., 2012) while ASYMMETRIC1 (AS1) and a kinase ERECTA are required for adaxialization in the leaf (Xu et al., 2003). All these gene families are upregulated in *PID^OE^* lines, with exception of the downregulation of *YAB1/FILAMENTOUS FLOWER* (*FIL1*), which suggests that PID might influence leaf lamina growth either directly or indirectly (**Figure 4**).

## Discussion

*PID^OE^* lines showed severe shoot growth phenotypes (Christensen et al., 2000) leading us to hypothesize that the cause and severity of the leaf phenotypes were due to affected auxin transport in the leaves, causing auxin retention in the top leaf blade because of changed polarity of PINs in the epidermal cells in the leaves. Previously, Zhao et al. (2001) reported elevated endogenous auxin levels and downward curled narrower leaves in the *yucca* mutants. Here we showed similar responses in *PID^OE^* lines and exploited the role of *PID^OE^* as auxin modulator. In our opinion these *PID^OE^* lines proved to be a good tool to study auxin dose-dependent processes. Such subtle changes in auxin levels are not easily achieved, and this provides a good opportunity to study their effect on development.

### *PID* Overexpression Lines Show Alterations in Auxin Homeostasis and Signaling

To validate our hypothesis of auxin involvement in growth defects in *PID^OE^* lines, we studied four different auxin related pathways; biosynthesis, conjugation, transport, and signaling. Since transport of auxin in the leaves can’t be assayed easily we relied on other indirect means/interpretations. IAA is the most abundant form of auxin in vascular plants including Arabidopsis. Free IAA is the active form of auxin and represents only a small percent of the total IAA, which mostly includes IAA conjugated to sugars, amino acids and peptides. *PID^OE^* lines showed high levels of both free and conjugated forms of IAA in the first pair of leaves compared to the WT leaves (**Figure 2**). Auxin regulates leaf blade and cell expansion in a dose-dependent manner, where supraoptimal concentrations are inhibitory (Thimann, 1939; Evans et al., 1994; Keller et al., 2004). High auxin levels in *PID^OE^* lines could also affect growth in a similar manner. RNA-seq data showed downregulation of a YUC8 gene involved in auxin biosynthesis, while the expressions of other biosynthesis genes were unchanged. Both *PID^OE^* lines lack the characteristic auxin overproducing phenotypes, like the presence of plentiful lateral and adventitious roots, elongated hypocotyl and petioles, and epinastic cotyledons (Boerjan et al., 1995; Benjamins et al., 2001; Zhao et al., 2001). However, leaves were curled downward and the *PID^OE^* lines also show a lack of apical dominance, a phenotype often related to auxin homeostasis mutants (Estelle and Somerville, 1987; Christensen et al., 2000; Benjamins et al., 2001). Therefore, downregulation of auxin biosynthesis and upregulation of conjugation and deconjugation-related genes in the RNA-seq data indicate a feedback mechanism to maintain homeostasis between overloaded free IAA and conjugated forms. Overall, the total IAA concentration of P10 remained significantly higher than that of the WT, whereas in P21 the higher IAA concentration dropped to nearly WT levels when the tissues were mature. This result is consistent with the changes in the expression levels of *PID* in the P21 line similar to qPCR transcript abundance data, suggesting that a certain level of PID is required to maintain high auxin levels.

PIN localization determines the direction of auxin flow in plants (Wisniewska et al., 2006). Extensive research proves that PID regulates auxin fluxes by regulating subcellular localization of PIN proteins, where *PID* overexpression causes apicalization of PIN protein at the cellular membranes in various cells in Arabidopsis, including the leaf epidermal cells (Friml et al., 2004; Kaplinsky and Barton, 2004). This apical polarity bias causes upward (shootward) auxin flow, compromising the auxin maxima at the root tip, which causes root meristem collapse (Benjamins et al., 2001; Friml et al., 2004; Armengot et al., 2016). Partial rescue of root phenotypes in *PID^OE^* lines after application of an auxin transport inhibitor, NPA (N-1-Naphthylphthalamic Acid) further supports this statement (Benjamins et al., 2001). Here, we propose that apical PIN polarity in the leaves causes auxin retention, especially in the top leaf region (**Figures 2, 3**), which could have disturbed the source to sink relationship between shoot and root causing various developmental defects. This problem of drainage or efflux caused by PINs provides a reason for the accumulation of auxin in the top marginal parts of the leaves, at least partially, if not entirely, since PID only transiently phosphorylates PINs, and the proportion of PID co-localize with PIN at the plasma membrane is inversely related to the PID concentration (Barbosa et al., 2014; Fozard et al., 2013). Additionally, PID also phosphorylates ABCB1 and thus affects its auxin efflux function as well (Henrichs et al., 2012), so its not clear to what extent the leaf phenotypes reported in *PID^OE^* lines can be directly attributed to PIN proteins alone. Nevertheless, IAA measurements and visualization of auxin signaling point to a failure of auxin export from the leaves in the *PID^OE^* lines. Wild type plants treated with NPA mimicked this genetic effect by blocking the auxin transport and similarly also showed a clear reduction in rosette growth with increasing concentrations (Supplementary Figure 6). As mentioned earlier increased efflux from the root tips causes root meristem collapse since PID enhances efflux of auxin from the cells (Benjamins et al., 2001; Friml et al., 2004; Lee and Cho, 2006; Zourelidou et al., 2014). According to Benjamins et al. (2001), effect of the primary root meristem collapse by *PID* overexpression is later compensated by the formation of lateral roots, that start to act as new auxin sinks. This is plausible especially in P21 where the formation of lateral roots (Benjamins et al., 2001) may have resulted in changes in auxin distribution between the shoot and roots. This could additionally explain the lowering of auxin levels in the leaves in later developmental stages in P21. P10, on the other hand, showed very few lateral roots (Benjamins et al., 2001) and thus, accordingly, maintained high auxin in the leaves. From these experiments, it can be concluded that PID altered the auxin flow in the *PID* overexpressing plants.

Auxin signaling reporters in the *PID^OE^* lines (*DR5::GUS* and *DR5_rev_::GFP*) support the accumulation of auxin (**Figure 2**) and changes in auxin response in the leaves (**Figure 3**). Since DR5 reporter and thus auxin response is different between leaves and roots, it cannot be said if PID is a negative or positive regulator of auxin signaling or if perhaps the effect is tissue-specific. Moreover, PID is previously implicated to affect auxin signaling based on the presence of overlapping phenotypes between *pid* mutants and mutants of *MP (ARF5), ETTIN (ARF3)*, and *SHORT HYPOCOTYL 2 (IAA3)* (Christensen et al., 2000). Upregulation of *MP*, and *ARF3* in *PID^OE^* lines besides other ARFs and Aux/IAAs in our RNA-sequencing data also suggest a link.

### *PID* Overexpression May Affect Leaf Growth by Perturbing Cellular Processes

Single *pid* knockout mutants showed only marginal phenotypic alterations in their leaves (Bennett, 1995: this work). This may be due to functional redundancy between PID and its homologs (Cheng et al., 2008). On the other hand, kinematic growth analysis showed that the growth in *PID^OE^* was restricted throughout development and due to inhibited cell division and cell expansion. Six different processes occurring during leaf development influence its final size: the number of cells recruited from the shoot apical meristem at the time of primordium initiation, duration of cell division and expansion, the rate of cell division and expansion, and finally meristemoid division in the cells of the stomatal lineage (Gonzalez et al., 2012). *PID^OE^* also displayed a shorter cell proliferation phase (more so in P21), however, the duration of the expansion phase seems not to be affected, as all three genotypes reached cell size maturity from 24 DAS onward. Reduction in stomatal index points toward problems in meristemoid division as well. On top of this, cell division and expansion rates are clearly affected in both *PID^OE^.* Since *PID^OE^* affected multiple processes, contributions of individual cellular process underlying the phenotype become difficult to pinpoint. Since (1) P21 had a more drastic reduction in cell number than P10 due to the higher *PID* overexpression at that time and (2) P10 showed a smaller cell area at maturity compared to P21, when a decrease in the *PID* transcript levels were noticed in P21, it can be said that the severity to both processes is dependent on *PID*-dosage and its resulting effects on auxin levels.

### Transcription Data Present Insights into Cellular and Organ Level Phenotype

RNA sequencing provided insights into the mechanisms behind the phenotype. However, there was little overlap between the two-overexpression lines (**Figure 7A**). This could be partially due to the differences in *PID* expression levels and thus auxin levels at the mentioned time points of the sampling for the RNA-sequencing and partly to the fact that even transient changes in auxin affect a plethora of events at the cellular and molecular level. This is evidenced by the affected functional categories in PageMan ontology tool in our data (**Figure 8**) and in previous reports (Nemhauser et al., 2006; Paponov et al., 2008). This being superimposed on changes on developmental rates of the organ, involving large transcriptional reprogramming (Beemster et al., 2005), can explain the observed differences between the two lines.

Variations in transcript levels of several leaf growth and development-related genes were detected in *PID^OE^* lines and they correlatively suggest a role of PID in defining leaf form and size. Cell sizes and leaf growth are often correlated with cellular DNA content (Donnelly et al., 1999; Massonnet et al., 2011). The P21 line showed changes in endoreduplication index (EI) over time, and this can also be coupled/related to changes in leaf (and cell size) growth and the *PID* expression levels at that time. High auxin levels suppress the progression of endocycles, and is also coupled to the progression of cell differentiation, which is marked by a sudden increase in cell size (De Veylder et al., 2007; Ishida et al., 2010). This is also vividly evident in P10 since it has a reduced endoreduplication index and cell area. P21, on the other hand, showed the opposite scenario because of the lower auxin levels compared to P10 when approaching maturity.

## Conclusion

Our results provide an insight into the influence of PID on auxin levels and distribution. PID is known as positive regulator of PAT. Here we showed that PID via its effect on auxin accumulation and distribution might also indirectly influence auxin metabolism and signaling and thus indirectly have a regulatory effect on leaf development as well.

## Author Contributions

KS planned and performed most of the experiments, analyzed the data, and wrote the article with contribution from EP, GB, TB, LV, and KV. MM performed next generation sequencing. MZ and DB provided assistance with crossing and genotyping experiments. EP, GB, and KV conceived the project and KV supervised the research.

## Conflict of Interest Statement

The authors declare that the research was conducted in the absence of any commercial or financial relationships that could be construed as a potential conflict of interest.

## Footnotes

1 http://rsbweb.nih.gov/ij/

2 www.arabidopsis.org

3 https://www.r-project.org/
