# Supplementary material for: Alteration in Auxin Homeostasis and Signaling by Overexpression Of PINOID Kinase Causes Leaf Growth Defects in Arabidopsis thaliana
Source: Front Plant Sci. 2017 Jun 14;8:1009. doi: 10.3389/fpls.2017.01009 (PMC5470171; doi:10.3389/fpls.2017.01009)
Supplement: Supplementary file 3 [file Data_Sheet_3.pdf]

Supplementary Material

Alteration in auxin homeostasis and signaling by overexpression of PINOID kinase causes leaf growth defects in *Arabidopsis thaliana*

Kumud Saini, Marios Nektarios Markakis, Malgorzata Zdanio, Daria Balcerowicz, Tom Beeckman, Lieven De Veylder, Els Prinsen, Gerrit T.S. Beemster, Kris Vissenberg*

***Correspondence**: **Kris Vissenberg** kris.vissenberg@uantwerpen.be

1. Supplementary data

Supplementary Data 1 RNA sequencing data for all the genes for which expression was detected in all three replicates of at least one timepoint and genotype i.e. WT and two *PID* overexpression lines.

Supplementary Data 2 RNA sequencing data for all 3805 differentially expressed genes in at least one timepoint and genotype i.e. WT and two *PID* overexpression lines.

2. Supplementary Figures and Tables

2.1 Supplementary Figures

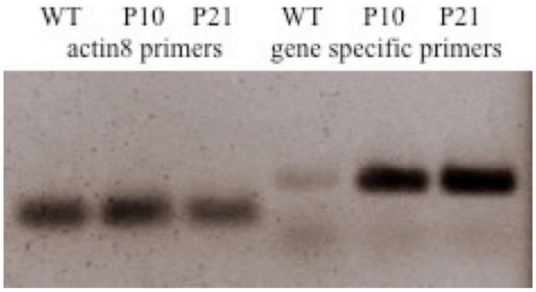

Supplementary Figure 1 *PID* expression in overexpressing lines. RT-PCR for *PID*^{OE} lines and WT using cDNA from the first pair of leaves at 9 DAS.

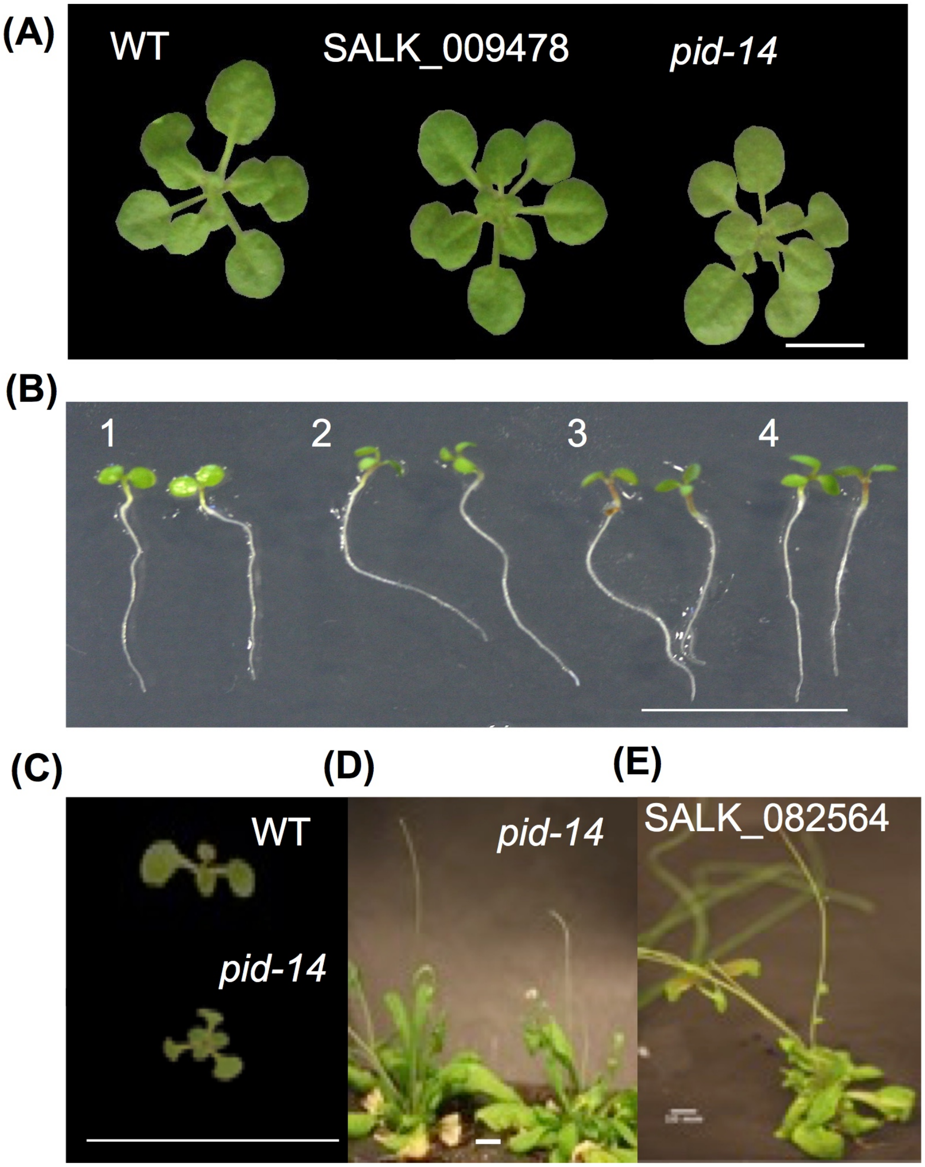

Supplementary Figure 2 Phenotypes of Arabidopsis *pid* mutants. Pictures of 22 DAS old rosettes of WT, SALK_009478 and *pid-14* (A). Pictures of young seedlings with occasional three cotyledons − WT, *pid-14*, SALK_009478, SALK_082564 from 1- 4 in the same order (B). Cotyledon comparison between WT and *pid-14* at 12 DAS (C) *pin*-like inflorescence of *pid-14* (D) and SALK_082564 (E). Scale bar =10 mm.

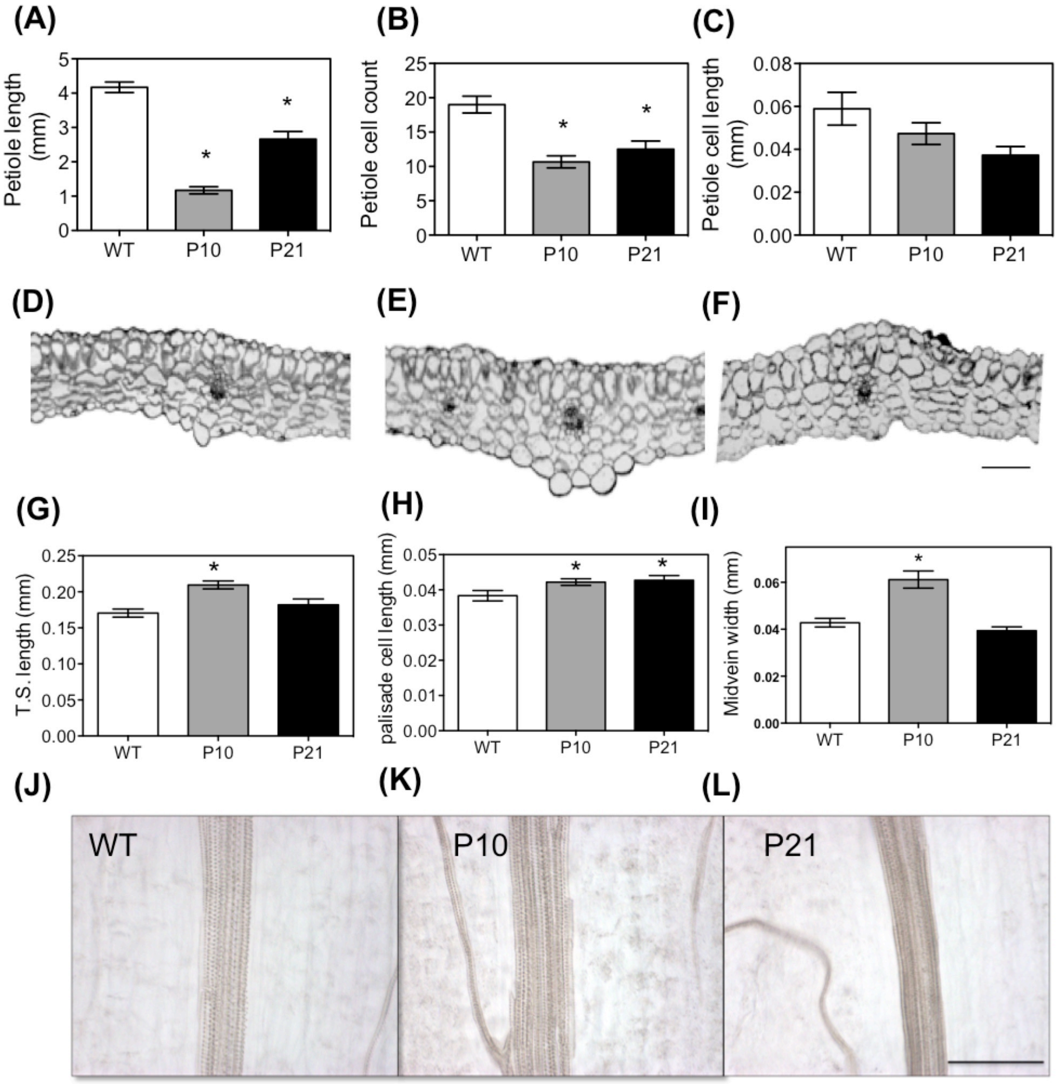

Supplementary Figure 3 Phenotypes of Arabidopsis *PID^{OE} lines*. Petiole-related measurements in WT and *PID* overexpression lines (A-C). Transverse section of leaves at 16 DAS - WT (D), P10 (E) and P21 (F). Graphs with width of transverse section along the midrib and length of spongy palisade cells (G,H). Midvein width comparison between genotypes (I) Midvein width comparison between WT (J), P10 (K) and P21 (L). Asterisks indicate significant differences with the wild type. Error bars represent ± SE (t-test, $P < 0.05$). Scale bar =10 mm.

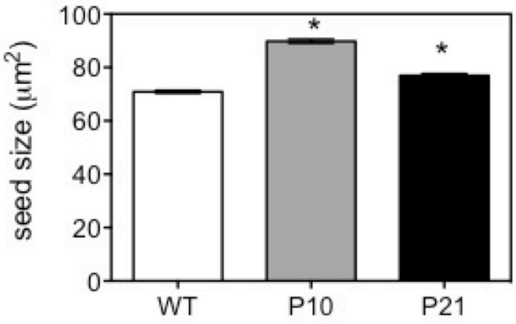

Supplementary Figure 4 Seed size comparison between WT and *PID* overexpression lines. Asterisks indicate differences with WT. Error bars ± SE (t-test, $P < 0.01$); n=500-600

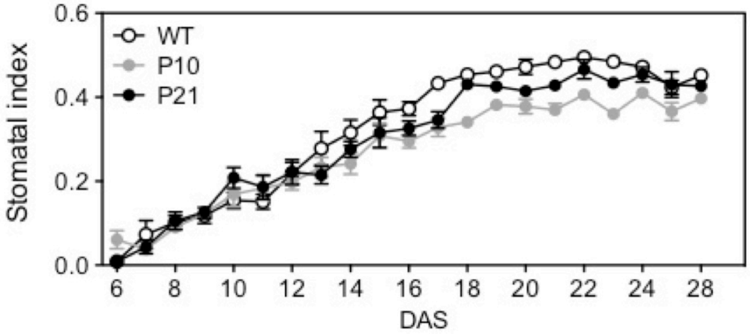

Supplementary Figure 5 Stomatal index comparison between WT and two *PID* overexpression lines. Stomatal index was measured throughput development of the first leaf pair. Error bars represent averages ± SE; n=6

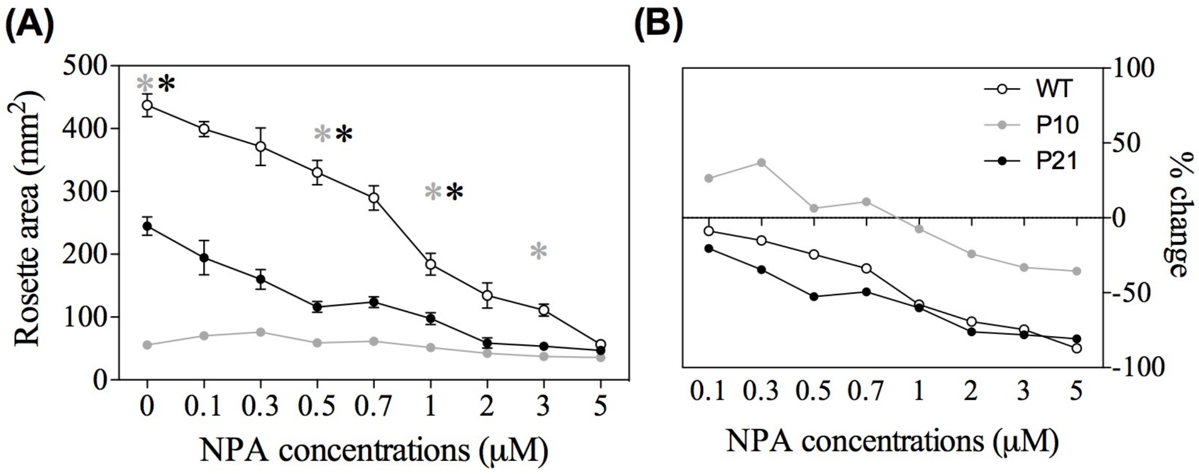

Supplementary Figure 6 Influence of auxin transport inhibition on leaf growth. Effect of different concentrations of NPA at 22 DAS on rosette area (A) and corresponding % change in growth (B) in WT, P10 and P21. Differences with respective control (0 NPA) are shown as percent change. Grey and black asterisks represent significant difference of P10 and P21, respectively, compared to the WT. Symbols are averages ± SE (t-test, $P < 0.05$); n=60-80.

2.2 Supplementary tables

Supplementary Table 1 Primer sequences

**SALK_082564**	TCCGTCATAGACAACCTCACC	GAGTAAGCGTACGAATGAGCG
**SALK_009478**	CTTTCATCGCAAAATACGAGC	CCAATCATCTTGAATTAGCCAG
**SALK_049736 (*pid-14*)**	CAGTCGGGAAACTCAACTGTC	ATTTTGCGATGAAAGTTGTGG
**LBb1.3**	ATTTTGCCGATTTCGGAAC	
**Actin8**	TGCTGGTCGTGACCTTACTG	CAATTTCCCGTTCTGCTGTT
***PID* intron spanning primer for RT and qPCR**	GAGCAGAGATGGAGAAAACGA	TGAAAATGCTTGACCATCCA

Supplementary Table 2 Differentially expressed cell division related genes between WT and *PID* overexpression lines. Significant log2 fold changes are marked as boldface. Parenthesis denotes days after stratification (DAS).

Functional category	Feature ID	Name	P10 (9)	P21(9)	P10 (16)	P21 (16)
cell cycle	AT2G27970	CKS2	-0.285	**-0.990**	0.678	0.576
cell cycle	AT1G80370	CYCA2;4	-0.283	-0.450	**-1.897**	-0.684

cell cycle	AT2G26760	CYCB1;4	-0.279	-0.635	**0.910**	0.525
cell cycle	AT4G35620	CYCB2;2	-0.748	**-1.226**	-0.141	-1.222
cell cycle	AT4G37630	CYCD5;1	-0.298	-0.144	**-1.498**	0.060
cell cycle	AT5G27620	CYCH;1	-0.333	-0.534	**-1.057**	-0.063
cell cycle	AT2G23430	KRP1	0.600	**1.136**	-0.342	-0.318
cell cycle	AT3G50630	KRP2	0.011	0.579	**0.837**	-0.235
cell cycle	AT5G48820	KRP3	**-0.913**	**-1.485**	-0.336	0.608
cell cycle	AT1G49620	KRP7	0.078	-0.712	**-1.644**	-0.148
cell cycle	AT2G27970	CKS2	-0.285	**-0.990**	0.678	0.576
E2F/DP TF	AT2G36010	E2FA	-0.232	-0.725	**-1.698**	0.486
E2F/DP TF	AT1G47870	E2FC	0.531	0.293	**-0.824**	-0.040
E2F/DP TF	AT3G01330	DEL3	-0.725	**-1.451**	-1.367	-0.135
DNA synthesis/chromatin structure	AT5G16690	ORC3	-0.869	**-1.243**	-0.016	0.891
cell division	AT3G48150	APC8	-0.368	**-1.278**	0.569	0.695
cell division	AT2G18290	APC10	-0.968	**-1.00**	**-1.217**	-0.553
protein postranslational modification	AT1G02970	WEE1	-0.775	**-1.497**	-0.410	0.427
protein degradation ubiquitin.E2	AT1G50490	UBC20	-0.205	**-1.001**	0.282	0.680
RNA regulation of transcription	AT4G11920	CCS52A2	0.486	**0.839**	0.978	1.087

Supplementary Table 3 Differentially expressed cell expansion-related genes between WT and *PID* overexpression lines. Significant log2 fold changes are marked as boldface. Parenthesis denotes

days after stratification (DAS).

**Functional category**	Feature ID	Name	P10 (9)	P21(9)	P10 (16)	P21 (16)
cell wall precursor synthesis	AT1G12780	UGE1	-0.765	-0.501	**-1.490**	-0.606
	AT4G23920	UGE2	0.273	0.691	**1.367**	0.035
	AT3G02570	MEE31	0.431	**0.808**	0.343	0.191
cell wall synthesis	AT5G03760	ATCSLA09	0.372	**0.935**	**0.784**	0.312
	AT5G16190	ATCSLA11	0.524	0.709	-0.884	**-1.728**
	AT4G13410	ATCSLA15	-0.146	0.516	1.389	**3.106**
	AT4G31590	ATCSLA05	0.151	0.254	**0.797**	0.300
	AT1G24070	ATCSLA10	1.178	**2.298**	**5.279**	0.566
	AT3G28180	ATCSLA04	0.377	**0.890**	-0.497	-0.357
	AT4G24010	ATCSLAG1	-0.266	-0.439	**1.815**	-1.218
	AT4G23990	ATCSLAG3	0.596	0.607	**1.825**	-0.608
	AT5G44030	CESA4	0.292	0.745	**1.300**	0.327
	AT1G55850	ATCSLE1	0.472	**1.023**	**1.619**	-0.351
	AT1G02730	ATCSLD5	-0.118	**-0.819**	-0.146	-0.133
	AT1G23480	ATCSLAO3	-0.404	-0.411	**-1.407**	0.116
	AT2G03220	FT1	-0.085	0.220	**0.901**	0.133
	AT5G62620		0.401	**0.796**	**1.273**	-0.016
	AT2G20370	KAM1	0.737	**0.843**	**0.908**	0.085
	AT5G54690	GAUTL1	2.157	**2.887**	**1.458**	0.818

	AT1G19300	GALT1	0.472	0.245	**0.931**	-0.089
	AT5G33290	XGD1	0.540	0.734	**1.889**	-0.142
cell wall proteins	AT5G03170	FLA11	0.682	**1.066**	0.825	0.717
	AT5G55730	FLA1	0.253	0.279	**0.831**	0.646
	AT2G47930	AGP26	-0.088	0.325	**-1.561**	-0.350
	AT4G16980		0.133	0.355	**-1.188**	0.150
	AT5G44130	FLA13	-0.018	-0.001	**-0.795**	0.045
	AT4G12730	FLA2	-0.033	0.229	**-0.851**	0.071
	AT2G42800	AtRLP29	-0.214	**-1.593**	0.629	0.151
	AT3G24480		**1.324**	**1.385**	**-0.832**	0.075
	AT4G16790		0.804	**1.677**	-0.659	-0.414
	AT1G76930	ATEXT4	**-1.966**	-1.925	-1.759	2.742
	AT3G22440		0.269	0.546	**1.277**	0.045
cell wall degradation	AT1G22880	CEL5	-0.169	-0.211	-0.028	**-0.792**
	AT2G32990	AtGH9B8	1.320	1.785	**1.586**	0.529
	AT3G52840	BGAL2	-0.102	-0.013	**0.874**	-0.336
	AT5G64570	XYL4	-0.704	**-1.191**	0.031	0.554
	AT5G66460	ATMAN7	0.159	0.666	**2.421**	-0.242
	AT4G30270	MERI5B	-0.150	-0.067	**-3.653**	-0.405
	AT3G19620	ANAC001	**1.934**	**2.938**	**1.883**	-0.356
	AT1G58370	RXF12	1.443	0.775	**-2.034**	-0.168

	AT5G49360	BXL1	**-1.675**	**-2.372**	**-3.774**	-0.612
	AT1G02640	BXL2	**-1.370**	**-2.140**	-0.066	-0.030
	AT1G67750		0.442	**0.897**	**2.821**	**1.099**
	AT1G10640		0.321	0.666	**1.877**	**1.369**
	AT4G23820		-0.013	-0.327	-0.361	**0.787**
	AT5G14650		-1.067	-0.202	**1.504**	**1.250**
	AT1G56710		1.351	**2.360**	**2.008**	1.141
	AT5G49215		0.233	0.500	**-1.238**	0.368
	AT1G23460		-0.327	**1.002**	0.768	-0.393
	AT1G09890		0.954	0.841	**2.561**	1.054
	AT5G63180		0.722	**0.967**	-0.296	-0.071
	AT1G19170		-0.223	-0.019	**1.026**	0.145
cell wall modification	AT2G20750	ATEXPB1	0.826	**1.255**	**0.878**	**1.192**
	AT5G56320	ATEXPA14	-1.659	-0.744	**-2.201**	**-1.495**
	AT4G30290	ATXTH19	-0.899	-1.119	**-4.254**	**-2.082**
	AT4G28250	ATEXPB3	0.327	0.086	**2.198**	0.682
	AT3G44990	XTR8	0.283	**0.842**	**-1.485**	-0.331
	AT2G37640	ATEXPA3	-0.240	-0.402	**-0.787**	0.445
	AT3G55500	ATEXPA16	0.379	**1.565**	2.036	-0.847
	AT4G17030	ATEXLB1	0.478	**1.943**	**2.981**	0.219
	AT5G65730	XTH6	-0.499	0.190	**-2.228**	0.155

AT2G39700	ATEXPA4	0.112	0.372	**1.205**	0.172
AT2G06850	EXGT-A1	-0.374	0.108	**-0.882**	0.046
AT4G38210	ATEXPA20	-0.162	-0.310	**1.235**	-0.091
AT4G37800	XTH7	-0.300	**0.894**	-0.419	0.011
AT1G11580	ATPMEPCRA	0.737	0.213	**-0.949**	**-0.803**
AT5G53370	ATPMEPCRF	0.469	**1.578**	0.458	-0.156
AT1G53830	ATPME2	-0.422	-0.422	**-1.665**	-1.114
AT3G49220		0.278	0.365	**0.987**	0.110
AT5G47500	PME5	0.211	0.216	**1.794**	-0.208
AT1G41830	SKS6	0.590	**0.775**	-0.124	-0.033
AT3G43270		1.084	**1.557**	0.157	0.124
AT1G57590		-0.159	-0.710	1.959	**-2.307**
AT3G09410		0.230	0.174	**0.790**	-0.523
AT1G23200	HMS	1.546	**2.103**	**2.433**	-1.056
AT3G59010	PME35	**1.371**	**1.050**	**1.535**	0.175
